# Affective Science Research: Perspectives and Priorities from the National Institutes of Health

**DOI:** 10.1007/s42761-023-00218-w

**Published:** 2023-09-06

**Authors:** Janine M. Simmons, Andrew Breeden, Rebecca A. Ferrer, Arielle S. Gillman, Holly Moore, Paige Green, Vani Pariyadath, Erin B. Quinlan, Aleksandra Vicentic

**Affiliations:** 1 National Institute On Aging, Bethesda, MD USA; 2 National Institute of Mental Health, Bethesda, MD USA; 3 National Cancer Institute, Bethesda, MD USA; 4 National Institute On Minority Health and Health Disparities, Bethesda, MD USA; 5 National Institute On Drug Abuse, Bethesda, MD USA; 6 National Center for Complementary and Integrative Health, Bethesda, MD USA

**Keywords:** Perspectives, Funding Priorities, National Institutes of Health

## Abstract

Affective science is a broad and burgeoning field, and the National Institutes of Health (NIH) support research on a similarly broad range of topics. Across NIH, funding is available for basic, translational, and intervention research, including research in non-human animals, healthy populations, and those with or at risk for disease. Multiple NIH Institutes and Centers have specific programs devoted to topics within the affective science umbrella. Here, we introduce the funding priorities of these six: the National Cancer Institute (NCI), National Center for Complementary and Integrative Health (NCCIH), National Institute of Mental Health (NIMH), National Institute on Aging (NIA), National Institute on Drug Abuse (NIDA), and National Institute on Minority Health and Health Disparities (NIMHD). We then discuss overlapping themes and offer a perspective on promising research directions.

The experience, expression, and regulation of emotions influence many facets of health and well-being. Affective states and affective regulation can mediate and moderate behavioral and physiological processes, thereby impacting health outcomes. Social disconnection and loneliness increase the odds of all-cause mortality by more than 25% and have been labeled an epidemic by the United States Surgeon General (Holt-Lunstad et al., 2015; Murthy, 2023). Stressful exposures are well-established risk factors for respiratory and cardiovascular disease, obesity and diabetes, mood, anxiety, and substance use disorders (Slavich, 2016). With COVID-19, a clear need has emerged for cross-cutting research on acute and chronic stress, loss and bereavement, social isolation and loneliness, interpersonal violence, and burnout among healthcare workers. Emotion dysregulation is a core symptom of many neurodegenerative diseases, psychiatric disorders, and traumatic brain injury (Beauchaine & Crowell, 2020; Weis et al., 2022; Wise et al., 2019). Affective states influence health-related decision making and behavior (Ferrer & Mendes, 2018), and training in specific emotion regulation strategies may improve weight loss in obese adolescents, mitigate the distress associated with cancer, improve functional outcomes for people with psychiatric disorders, and benefit caregivers of those with chronic health conditions (Hadley et al., 2020; Lincoln et al., 2022; O’Toole et al., 2019; Sloan et al., 2017; Smith et al., 2023). Emerging evidence also points to the beneficial health effects of positive emotions and the potential for interventions to improve health through increasing emotional well-being (Mroczek et al., 2015; Pressman et al., 2019; Pourtois et al., 2021; Villanueva et al., 2021). Here, we focus on the interests of six of the NIH Institutes and Centers that include topics in affective science in their strategic plans and priorities.

## National Cancer Institute (NCI)

Cancer control research seeks to lessen the burden of cancer on individuals, communities, and populations by reducing the incidence, morbidity, and mortality of cancer and improving the quality of life in cancer survivors through the systematic implementation of evidence-based interventions for prevention, early detection, diagnosis, treatment, and palliative care (Hiatt & Rimer, 1999)*.* Cancer is a particularly affective-laden health threat, and behaviors that confer or reduce risk are often rewarding or unpleasant (Ferrer et al., 2015). Cancer provides an ecologically valid context for understanding fundamental questions about affective processes, and applying foundational discoveries in affective science can yield tremendous benefits for cancer control.

Decisions about health and health behaviors confer significant risks and benefits along the cancer control continuum, which includes etiology, prevention, treatment, survivorship, and end-of-life (National Cancer Institute, Division of Cancer Control and Population Sciences, 2020). Association studies implicate affective states in cancer-related information processing, decisions about cancer risk and prevention behaviors, and decisions about treatment and treatment adherence. However, our understanding of why affect matters and how emotions influence single and multiple-event decisions relevant to cancer control is in its infancy. Examples of cancer-related affective science questions include, but are not limited to (1) how do biobehavioral processes of stress and loneliness contribute to cancer incidence and progression, and under which conditions is this likely to occur; (2) how do complex, blended, and dynamic affective states unfold over time to influence cancer risk behaviors; (3) how do expectancies about the future, and affective responses to these, influence decisions about cancer screening, treatment, palliative care, and end-of-life care; and (4) how do emotion co-regulation and emotional contagion contribute to the dynamics of and experiences with cancer caregiving?

NCI is committed to health equity and particularly interested in facilitating research that engages individuals from groups historically underrepresented in or excluded from biomedical and behavioral research. More information on the affective science research domains prioritized by NCI, as well as a selection of awarded affective science grants, can be found on the NCI website (National Cancer Institute, Behavioral Research Program, 2022). Investigator-initiated research project applications are encouraged.

## National Center for Complementary and Integrative Health (NCCIH)

The mission of NCCIH is to determine the fundamental science, usefulness, and safety of complementary and integrative health approaches and their roles in improving health and healthcare within the framework of whole person health. Importantly, this is not limited to physical health, as psychological, affective, and social factors are key aspects of the whole person.

One related area of particular importance to NCCIH is emotional well-being (EWB). NCCIH seeks to fund research incorporating transdisciplinary approaches in the basic social, behavioral, psychological, and biological sciences to understand the factors that influence EWB, as well as clinical research on the use of mind–body interventions to enhance EWB and whole person health. NCCIH’s EWB High-Priority Networks include efforts to (1) explore the relationship between economic burden of illness and EWB; (2) study the modifiable elements of EWB across biological, behavioral, and experiential levels; and (3) deepen our understanding of how best to measure EWB and mechanisms through which mind–body interventions promote and maintain EWB. The EWB Networks recently published a provisional conceptualization of EWB, accompanied by commentaries from various well-being research stakeholders, including NIH (Park et al., 2022).

NCCIH extended its interest in emotional health in response to the growing youth mental health crisis exacerbated by the COVID-19 pandemic. Schools have increasingly become key access points of mental, emotional, and behavioral (MEB) health services for youth and have the capacity to reach large and, importantly, diverse populations of children at different developmental time periods. NCCIH supports projects to test the efficacy or effectiveness of mind–body interventions that can be delivered in a school-based setting or with students to promote MEB health and prevent MEB disorders among youth.

## National Institute of Mental Health (NIMH)

NIMH’s mission is to transform the understanding and treatment of mental illnesses, and research on the mechanisms underlying mood, affect, and their disruptions in mental illnesses is an integral part of NIMH’s basic and translational research portfolios. Basic neuroscience priorities include investigations of neurobiological mechanisms underlying affect, resilience, and vulnerability to stress in humans and animals. NIMH supports research on mood and anxiety disorders in humans, including diagnostic and transdiagnostic studies of the developmental trajectories, etiology, progression, risk factors, assessment, treatment, and prevention of mental illness associated with dysregulated mood and affect. A transdiagnostic framework within NIMH’s Research Domain Criteria (RDoC) initiative supports studies in affect-relevant domains of functioning, including arousal and regulatory systems, negative valence (threat and loss), positive valence (reward), and social processes. NIMH also aims to enhance support of research on affective processes and disorders that engages underrepresented study populations to understand mechanisms that contribute to mental health disparities.

Examples of affective science research areas of interest to NIMH include (1) understanding how distributed brain circuits dynamically represent multi-dimensional social-emotional information. (2) Understanding the neurobiological mechanisms underlying stress mediated changes in social-affective and cognitive processes (see Simmons, Winsky et al., 2021). (3) Understanding healthy and dysfunctional neurodevelopmental trajectories at the molecular, cellular, and circuit-levels, including characterizing periods of risk and resilience. (4) Utilization of innovative methods for quantification of affective behaviors to enhance knowledge of the neurobiology of mood and affect, increase understanding of proximal triggers for mood symptoms, and inform clinical decision making for mood and anxiety disorders.

For animal studies, NIMH prioritizes research on neurobiological mechanisms of mental health-relevant domains of function and behaviors, rather than studying animal behaviors in terms of emotions accessible only in humans by self-report or developing models “of” specific mental illnesses (see Table 2, NOT-MH-19–053). NIMH requires that investigators use an experimental therapeutics approach when developing psychosocial or biological interventions (Insel & Gogtay, 2014).

## National Institute on Aging (NIA)

NIA supports research to understand the aging process and conditions associated with growing older, emphasizes the experiences and processes that play a causal role in shaping trajectories of aging, and serves as the primary institute for Alzheimer’s disease and related dementias (AD/ADRD) research.

NIA seeks to elucidate the core biopsychosocial processes underlying changes in affective functions, the interactions between affective and cognitive processes, and the complex profiles of emotional adaptation and compensation across *normative* developmental trajectories of aging. Topics of particular interest include (1) impacts of early-life adversity on later life outcomes; (2) individual- and context-based variations in affective processing in mid- and late-life; (3) neural mechanisms underlying emotional changes with age; (4) social isolation and loneliness; and (5) impacts of optimism, purpose in life, compassion, and generativity on health and well-being across the lifecourse. In the context of AD/ADRD, NIA seeks to identify predictive psychological, behavioral, and biological markers for changes in emotional expression and regulation that may presage cognitive decline. We also support the development of strategies to detect, prevent, and treat affective and behavioral dysregulation in people living with dementia, including effects on caregiver health and well-being.

NIA encourages basic and translational affective science research in humans and/or appropriate animal models. NIA strongly encourages the application of psychometrically sound behavioral assessment approaches, and use of digital technology to measure affective, motivational, and social responses across time in “real-world” contexts. NIA provides expansive support for longitudinal studies and cross-cohort comparisons. All NIA research is expected to make use of the NIA Health Disparities Research Framework (Hill, et al., 2015). NIA prioritizes an experimental medicine approach to intervention development (Onken, 2022; Stoeckel et al., 2023).

## National Institute on Drug Abuse (NIDA)

NIDA’s mission is to advance science on substance use and substance use disorders (SUD). NIDA’s interest in supporting research on affective neuroscience relates particularly to factors that influence risk for and protection from development of SUD, as well as relapse and recovery. Priorities include (1) expanding from studies of specific circuits or unidimensional constructs to the full constellation of affective and behavioral dysfunction observed in SUD; (2) multi-level and multidimensional approaches to conceptualizing SUD — considering individual (e.g., neurobiology), social (e.g., peer networks; Table 1, PAR-21–352), and structural (e.g., neighborhood disadvantage; Table 1, RFA-DA-23–028) factors; (3) interactions between biology and the environment across the lifespan (and across generations) that influence the SUD trajectory; and (4) mechanistic computational modeling of affective behavior and neurobiology relevant to SUD.

**Table 1:** Active NIH Notices of Funding Opportunity (NOFOs)*

NOFOs**	Title	ICs	Activity code(s)*	Final receipt date
PAR-21–263	Computational Approaches for Validating Dimensional Constructs of Relevance to Psychopathology	NIMH	R01	11/1/2023
PAR-21–264	Computationally-Defined Behaviors in Psychiatry	NIMH	R21	11/1/2023
PAR-21–175, -176	Understanding and Modifying Temporal Dynamics of Coordinated Neural Activity	NIMH	R01, R21	2/5/2024
PAR-22–072	Measures and Methods to Advance Research on Minority Health and Health Disparities-Related Constructs	NIMHD, NIDCR, NIEHS, NIMH, NCI, NEI, NIA	R01	5/8/2024
PAR-21–288, -289	Utilizing Invasive Recording and Stimulating Opportunities in Humans to Advance Neural Circuitry Understanding of Mental Health Disorders	NIMH	R01, R21	6/16/2024
PAR-21–349, -350, -352	Research on Biopsychosocial Factors of Social Connectedness and Isolation on Health, Wellbeing, Illness, and Recovery	OBSSR, NIA, NIAAA, NIDA, NINR, NCCIH, NCI	R01	9/8/2024
PAR-21–271	The Role of Work in Health Disparities in the U.S	NIMHD, NIA, NIDA, NIEHS, NIMH, NCI, NICHD	R01	9/8/2024
PAR-22–066, -067	Basic Neurodevelopmental Biology of Circuits and Behavior	NIMH	R01, R21	10/16/2024
PAR-22–064	Patient-Clinician Relationship: Improving Health Outcomes in Populations That Experience Health Care Disparities	NIMHD, NIAMS, NIDCD, NEI, NCI	R01	1/8/2025
PAR-21–358	Risk and Protective Factors of Family Health and Family Level Interventions	NIMHD, NINR, NCI, NIAAA	R01	5/8/2025
RFA-AG-24–025, 026	Leveraging Social Networks to Promote Widespread Individual Behavior Change	NIA, NCI	R01, R34	11/3/2023
RFA-DA-22–037, -038	Accelerating the Pace of Drug Abuse Research Using Existing Data	NIDA	R01, R21	11/15/2024
RFA-DA-23–028	NIDA REI: Research on Neurocognitive Mechanisms Underlying the Impact of Structural Racism on the Substance Use Trajectory	NIDA	R61/R33	11/14/2024
RFA-DA-23–029	NIDA REI: Research at Minority Serving Institutions on Neurocognitive Mechanisms Underlying the Impact of Structural Racism on the Substance Use Trajectory	NIDA	R61/R33	11/14/2024

^*^NOFOs are associated with special review panels and/or set-aside funds. Updated NOFOs can always be found at the NIH Guide for Grants and Contracts (https://grants.nih.gov/funding/searchguide/index.html#/). **Companion NOFOs are listed together. NOFOs may be issued as: Clinical Trial Not Allowed, Clinical Trial Required, Clinical Trial Optional, or Basic Experimental Studies with Humans (BESH) Required (https://grants.nih.gov/policy/clinical-trials/specific-funding-opportunities.htm)

Recognizing the unique challenge of modeling human neuroaffective function using non-human species, NIDA supports rigorous approaches that are grounded in cross-species operationalizations of neuroaffective processes. Given there are key social factors (e.g., structural racism) that cannot be fully modeled in non-humans and challenges with aligning human neurobehavioral development with that of model species, we encourage investigators to consider modeling analogous social factors (e.g., competition for resources, predator threat, affiliative interaction), and considering homologies (and limits thereof) across humans and model species with respect to the neuroaffective system of interest.

Given the complexities surrounding SUD, we encourage longitudinal research designs, the use of large datasets (Table 2, NOT-DA-21–003), and/or a dynamic systems approach to data analytics, complemented by more focused investigations in smaller, controlled studies. In all cases, research needs to be grounded in the broader social context to prevent spurious biological determinants and potential for stigma (Laird, 2021; Shim, 2021; Simmons, Conley et al., 2021), and NIDA encourages training and coordinated, multi-disciplinary efforts (Table 1, RFA-DA-23–028, -029; RFA-DA-24–027) to address this need.

**Table 2:** NIH Notices of Special Interest*

NOSI number(s)*	Title	ICs	Expiration date
NOT-AG-21–012	Integrative Studies of Neural Mechanisms Underlying Fundamental Affective Processes in Aging	NIA	1/8/2024
NOT-AT-21–002	Promoting Research on Interoception and Its Impact on Health and Disease	NCCIH, NIA, NIDCR, NIDA, NIEHS, NIMH, NCI	5/7/2024
NOT-DA-21–003	Leveraging Longitudinal Studies in Animal Models to Identify Neural Mechanisms of Vulnerability and Resilience to Substance Use Disorder	NIDA	NA
NOT-MH-18–058	Notice of Information: NIMH's Interest in Areas of Stress Biology Research	NIMH	NA
NOT-MH-19–053	Notice of NIMH’s Considerations Regarding the Use of Animal Neurobehavioral Approaches in Basic and Pre-clinical Studies	NIMH	NA
NOT-MH-23–120	Notice of Special Interest (NOSI): The Neural Mechanisms of Multi-Dimensional Emotional and Social Representation	NIMH	1/8/2025

^*^Notices of Special Interest describe an Institute’s specific research area(s) of interest and provide links to associated NOFOs

## National Institute on Minority Health and Health Disparities (NIMHD)

The mission of the National Institute on Minority Health and Health Disparities (NIMHD) is to improve minority health and reduce health disparities through research including integrative biological and behavioral science, as well as community health, population sciences, clinical and health services research, and research on health systems and workforce development. Critically, research supported by NIMHD must include a focus on one or more of the following NIH-designated populations that experience health disparities in the United States: African Americans, Latinos/Hispanics, American Indians and Alaska Natives, Asian Americans, Native Hawaiians and other Pacific Islanders, less privileged socioeconomic groups, underserved rural populations, and sexual and gender minorities.

The NIMHD Research Framework (National Institute on Minority Health & Health Disparities, 2022) encourages projects that use approaches encompassing multiple domains of influence (e.g., biological, behavioral, sociocultural, environmental, physical environment, health system) and multiple levels of influence (e.g., individual, interpersonal, family, peer group, community, societal) to understand and address health disparities. Thus, research which explores affective processes at multiple levels and/or interactions is highly encouraged. Affective science-related research topics of interest to NIMHD include, but are not limited to (1) effects of chronic stress on physiological functioning (allostatic load) across the life course; (2) strategies for coping with adversity and chronic stress, including the impact of racism and discrimination on health, well-being, and health behaviors; (3) affective implications of climate change; (4) examination of culturally specific affective factors and their relationship to health outcomes; (5) impact of affective factors on healthcare engagement and patient-clinician relationships; and (6) understanding positive processes, including resilience, emotional well-being, and thriving among populations that experience health disparities. NIMHD does not typically prioritize studies using animal models.

## Discussion

**Fig. 1:**
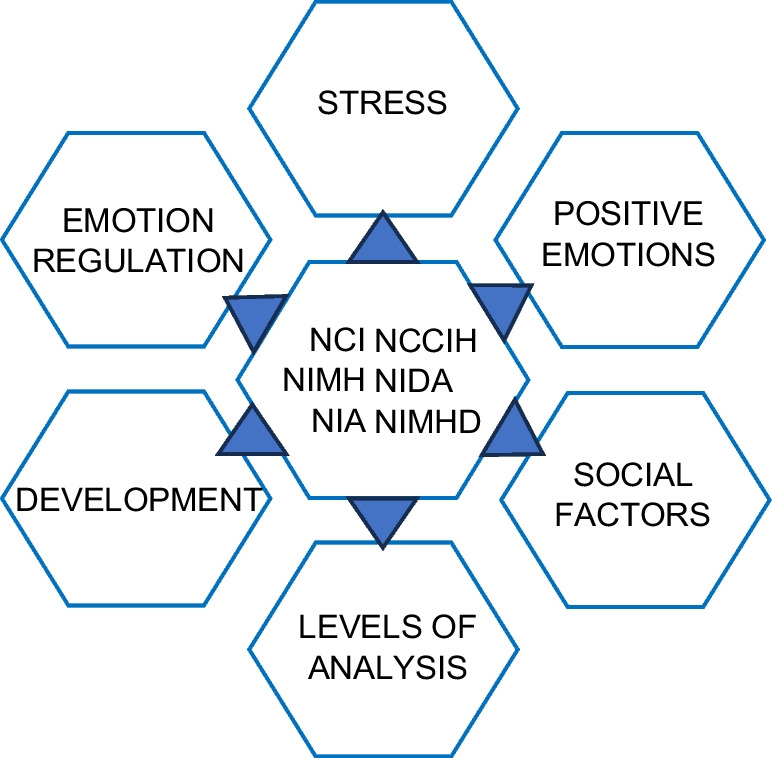
Cross-cutting themes in affective science priorities of the National Cancer Institute (NCI), National Center for Complementary and Integrative Health (NCCIH), National Institute of Mental Health (NIMH), National Institute on Aging (NIA), National Institute on Drug Abuse (NIDA), and National Institute on Minority Health and Health Disparities (NIMHD)

Although institute-specific health outcomes of interest vary across NIH, we emphasize here cross-cutting themes within affective science research that the authors of this commentary expect to guide future investments (Fig. 1).

### Stress

The larger NIH mission is to acquire knowledge to prevent, detect, diagnose, and treat disease and disability. One of the largest risk factors for disease and disability is stress, broadly construed. NIH Institutes will continue to prioritize fundamental research to understand how stress impacts both physiological and psychological systems. We also look forward to the continued development of innovative, effective, and broadly implementable ways to mitigate the adverse health effects of stressful experiences.

### Emotion Regulation, Emotion Buffering, and Resilience

NIH supports the development of strategies for individuals and communities to manage emotions, prevent adverse consequences of stress, and create conditions that promote recovery. Therefore, NIH priorities include studies of emotion regulation, and consideration of how individual, cultural, and contextual factors influence the appropriateness of specific emotion regulation strategies. Given health and disease involve more than one individual, NIH interests include questions of how interpersonal relationships and community structures buffer emotions. NIH institutes have joined forces to enhance a common agenda for resilience research, focusing on how biopsychosocial systems recover, grow, adapt, or resist perturbation from a stressor (Brown et al., 2023).

### Reward, Positive Emotions, and Emotional Well-Being

In addition to their direct effects in substance use disorders, rewards can indirectly impact health outcomes by shaping health behaviors and health-related decision-making (Shiota et al., 2021). Therefore, NIH continues to prioritize research on reward and on ways to safely and effectively target reward systems to reinforce health-promoting activities. NIH institutes also recognize that positive emotions may have benefits that go beyond the momentary (Pourtois et al., 2021), and increasingly support studies to test how positive states or traits may independently predict health outcomes. Similarly, NIH institutes are actively involved in initiatives to increase understanding of emotional well-being, as a health goal in and of itself (Feller et al., 2018; National Center for Complementary and Integrative Health, 2018; Park et al., 2022).

### Social and Interpersonal Factors

As the COVID-19 pandemic made abundantly clear, affective experiences depend upon social and interpersonal factors. NIH institutes will continue to prioritize fundamental research on processes associated with affiliation and prosocial emotions, as well as social threat and conflict. We encourage studies of how affective processes unfold between individuals and within groups, including emotion co-regulation, emotion contagion, and affective responses to social structural factors. We have expanded programs focused on the short- and long-term consequences of social connection, social isolation, and loneliness. We look forward to the development of interventions targeting improvements in health through social networks (Table 1, RFA-AG-24–025, -026).

### Levels of Analysis

NIH institutes emphasize integration of multi-level, interdisciplinary approaches to the affective sciences. Factors that influence affect reactivity and regulation range from genetic to environmental and everything in between. More inclusive descriptions of these multiple levels of analysis can be found in the RDoC matrix, NIA Health Disparities Research Framework, and NIMHD Research Framework (Cuthbert, 2022; Hill, et al., 2015; National Institute on Minority Health & Health Disparities, 2022). Importantly, NIH institutes seek to support both top-down and bottom-up investigations across levels of analysis. These studies require collaborations across disciplines, and future advances are likely to emerge from studies incorporating multi-level computational modeling and systems biology.

### Developmental Trajectories

In addition to these affective dimensions, time is always a factor. Exposures to stressors and rewards, social relationships, and emotional regulatory abilities change over time. Responses to events depend upon their timing, on (neuro)developmental sensitive periods, and on systemic capacities for change. For this reason, NIH institutes prioritize studies incorporating developmental trajectories and recommend the use of longitudinal designs and datasets. We also note that temporal scales vary enormously: molecular events unfold in nanoseconds; an individual’s developmental trajectory can take days, months, or years; affective and social experiences can influence health trans-generationally. NIH institutes recognize the challenges associated with incorporating the temporal dimension, and we encourage investigators in the affective sciences to embrace these challenges.

## Conclusion

All the NIH Institutes in this commentary support basic, translational, intervention, and clinical affective science research. NIH recognizes that affect is embedded with many aspects of life and health, and an NIH-wide goal is to better understand the roles and mechanisms by which emotional experiences impact health and well-being. We especially encourage multi-level investigations of stress, emotion regulation and buffering, resilience, reward systems, positive emotions and emotional well-being, and social and interpersonal factors and their impact during development and across the lifespan.
